# Trajectories of self-reported physical activity and predictors during the transition to old age: a 20-year cohort study of British men

**DOI:** 10.1186/s12966-017-0642-4

**Published:** 2018-02-07

**Authors:** Daniel Aggio, Efstathios Papachristou, Olia Papacosta, Lucy T. Lennon, Sarah Ash, Peter H. Whincup, S. Goya Wannamethee, Barbara J. Jefferis

**Affiliations:** 1 UCL Department of Primary Care & Population Health, Institute of Epidemiology and Health Care, UCL Medical School, Rowland Hill Street, London, NW3 2PF UK; 2 UCL Physical Activity Research Group, London, UK; 3 Department of Psychology & Human Development, UCL Institute of Education, London, WC1H 0 AA UK; 4 Population Health Research Institute, St George’s University of London, Cranmer Terrace, London, SW17 0RE UK

**Keywords:** Physical activity, Ageing, Retirement, Cardiovascular disease

## Abstract

**Background:**

Maintenance of physical activity (PA) during later life is associated with optimal health; however, the long-term trajectories of PA into old age and their predictors have not been extensively researched using latent class methods. This study aimed to identify trajectories of self-reported PA and their predictors in men transitioning from midlife to old age.

**Methods:**

7735 men (aged 40–59 years) recruited in 1978–80 were followed up after 12, 16 and 20 years, reporting PA, health status, lifestyle behaviours and socio-demographic characteristics. Group-based trajectory modelling identified the trajectories of PA and associations with time-stable and time-varying covariates. We considered a range of sociodemographic and health and lifestyle factors as potential covariates.

**Results:**

4952 men (mean baseline age 49.1 ± 5.6 years) providing PA data at ≥3 time points were included in analyses. Three distinct 20-year trajectories were identified: low decreasing (24.6%, *n* = 1218), light stable (51.1%, *n* = 2530) and moderate increasing (24.3%, *n* = 1204). Being older, having a manual occupation, having never married or had children, residing in the midlands or North of England, suffering from a range of health conditions, being a smoker/ex-smoker and never consuming breakfast cereal or alcohol were independently associated with reduced odds of belonging to the moderate increasing trajectory group compared to the low decreasing group. Of the time-varying covariates considered, leaving employment was associated with a decrease in PA in the low decreasing group (β −0.306, *p* < 0.001) but an increase in the light stable (β 0.324, *p* < 0.001) and moderate increasing groups (β 0.847, *p* < 0.001). Developing cardiovascular-related conditions was associated with a decrease in PA in the low decreasing (β −0.408, *p* < 0.001) and light stable groups (β −0.118, *p* < 0.001) but no association was observed in the moderate increasing group (β −0.060, *p* = 0.313).

**Conclusions:**

Three distinct trajectories of PA were identified in men transitioning from midlife to old age, of which nearly a quarter had persistently low levels of PA. Promotion efforts may need to focus attention prior to middle age and consider a number of sociodemographic, health and lifestyle factors to sustain PA into old age. The effects of retirement and development of cardiovascular disease may vary according to PA trajectories.

**Electronic supplementary material:**

The online version of this article (10.1186/s12966-017-0642-4) contains supplementary material, which is available to authorized users.

## Background

There is extensive evidence on the benefits of physical activity (PA) in older adults, including reduced risk of a number of diseases [1, 2], mortality [3–5], falls [6] and cognitive [7] and functional decline [8]. Despite the benefits, PA levels decrease rapidly in old age [9]. Maintenance of a physically active lifestyle during later life is associated with optimal health benefits [10–12]; however, very few studies have examined the trajectories of PA during the transition to old age to understand when declines occur and the factors that predict these changes [13–15]. Longitudinal studies investigating the determinants of change in PA in older adults have identified a number of factors associated with maintenance of PA. Sociodemographic factors such as female sex, lower education and income have been associated with reduced likelihood of being persistently active in older adulthood [13, 14]. Health status is also an important factor related to changes in PA in older adults. Poorer self-rated health, arthritis, depression, obesity and lower physical function are associated with declining PA [13, 16–18]. PA is known to protect against cardiovascular disease (CVD) [1], but the impact of developing CVD on subsequent long-term PA patterns remains unclear. Research on the effects of other life events coinciding with older age, such as retirement, on PA has produced mixed findings. There is evidence that older adults who stay employed are more physically active due to work-related travel [19], whereas other studies suggest that PA increases on retirement due to increased leisure time PA [20–23].

Although there is accumulating evidence relating these factors to changes in PA in older adults [13– 20, 22, 23], some longitudinal studies are limited by their statistical approach. Traditional growth curve analysis is often used to describe an average pattern of change over time, making the assumption that all individuals come from the same population and share a common trajectory. This approach may be unrealistic and does not allow for the possibility of different or unusual underlying patterns of change among participants. Furthermore, some studies assume specific trajectories of PA prior to analysis using researcher-driven or subjective classifications [16] rather than allowing for appropriate trajectories to emerge from the data itself. Subjective approaches may fail to identify important underlying subgroups with unusual patterns of behaviour change. Unlike the aforementioned approaches, group-based trajectory modelling (GBTM) is a data-driven method for identifying the unobserved heterogeneity in behaviour change and subsequently a number of distinct subpopulations that follow a similar pattern of behaviour over time [24]. This approach has been used frequently in studies of younger populations [14, 25–29] but has only recently been adopted in older adults [15]. More research utilising this approach in other older adult populations is required to fully understand the trajectories of physical activity between midlife and old age and, further, the factors that may be related to more favourable PA trajectories into later life. Based on current evidence in the literature, we hypothesised that a range of sociodemographic and health and lifestyle factors would be associated with PA trajectories into old age.

The aims of this study were firstly to identify distinct trajectories of self-reported PA from midlife to old age using GBTM methods. Secondly, to identify independent predictors of trajectory class membership and the effects of time-varying covariates (such as onset of CVD) on PA trajectories.

## Methods

### Participants

Data were drawn from the British Regional Heart Study, an ongoing prospective cohort study following up 7735 men recruited from primary care practices in 24 towns in Great Britain between 1978 and 80 when aged 40–59 years [30]. Men were followed up after 12, 16 and 20 years, completing a lifestyle and medical history questionnaire at each follow up and attending physical examinations at baseline and 20-year follow up. Participants provided informed written consent to the investigation. Ethical approval was obtained from the National Research Ethics Service Committee London.

### Measures

#### Self-reported PA

At all follow ups, participants self-reported their usual PA levels. Questions included how much time was spent on all forms of walking, time spent on recreational activities (such as recreational walking, gardening, chores and do-it-yourself activities) and engagement in sport/exercise. The same questionnaire was administered at each follow up to facilitate comparison between time points. Responses to each type of PA were scored based on the intensity and frequency of the activity [31, 32]. For example, making no journeys by foot was scored as 0 and >90 min/weekday was scored as 5. Scores were also heavily weighted for vigorous activities. For example, playing sport 4–7 times a month was given a score of 8. Scores for each item were summed together to give a total PA index. The original scoring system has been reported in detail elsewhere [33]. The total PA index was then used to group men into six categories (0–5); inactive; occasional (regular walking or recreational activity only); light (more-frequent recreational activities, sporting exercise less than once a week, or regular walking plus some recreational activity); moderate (cycling, very frequent weekend recreational activities plus regular walking, or sporting activity once a week); moderately vigorous (sporting activity at least once a week or frequent cycling, plus frequent recreational activities or walking, or frequent sporting activities only); or vigorous (very frequent sporting exercise or frequent sporting exercise plus other recreational activities). The 6-point total PA score was used for the analysis. These PA scores have previously been validated against heart rate and forced expiratory volume in 1 s [33] and more recently against objectively measured PA [34].

### Other measures

Variables that were not measured at each follow-up could only be considered as time-stable variables for the current analyses. Variables that were measured repeatedly at each follow up could be considered as time varying. However, a number of factors were considered before incorporating them as time varying in the model, including how much they changed over time, whereby those that had little variability over time were instead treated as time stable; whether the effects were different between trajectory groups, which could be investigated using a Wald test; and whether a change in the variable of interest represented an important life event that could in theory initiate change in PA.

#### Time-stable measures

At baseline men reported a number of sociodemographic characteristics, health conditions and lifestyle behaviours. These variables included current or longest held occupation (manual or non-manual); marital status (single, married or widowed/divorced); number of children (none or ≥1); doctor-diagnosed health conditions including arthritis, bronchitis and high blood pressure; other health problems including breathlessness and chest pain on exertion; body mass index (BMI) (normal weight: BMI <25.0 Kg/m^2^ or overweight/obese: BMI ≥25.0 Kg/m^2^); smoking status (current/ex-smoker or non-smokers); alcohol consumption (none, occasional [<1 drink/week], light [1–15 drinks/week], moderate [16–42 drinks/week] or heavy [>42 drinks/week]); region of residence (Scotland, North, Midlands and South); and weekly breakfast cereal consumption (none, occasional [1–2 times/week] or regular [>3 times/week]). Breakfast cereal consumption was used as an indicator of breakfast habits.

#### Time-varying measures

Number of CVD diagnoses and employment status were treated as time-varying variables*.* At each follow up men reported doctor diagnosis of CVD (stroke, heart attack, myocardial infarction, coronary thrombosis or angina). The number of CVD diagnoses was summed at each time point. Men also reported employment status at each follow up, from which men were classified as in employment (0) or not in employment (1). Years of follow up were derived from the date of survey completion. We considered using BMI and marital status as time-varying covariates; but due to high collinearity across assessments (variance inflation factor > 3) and because the effects of BMI were similar across all trajectory groups, these variables were instead treated as time-stable predictors. Data on alcohol consumption and smoking were also available at each follow up so were eligible for inclusion as time varying. However, as the impact of these behaviours was not the primary focus of this study we included them as time stable predictors in the main analysis. We did however explore them as time-varying covariates in supplementary analyses. All predictor variables were selected a priori based on evidence in the current literature and were hypothesised to be associated with PA levels.

### Data analysis

We used a GBT model to identify latent homogenous groups of study members with similar PA trajectories over 20 years by means of the Stata TRAJ plugin [35], which applies finite mixture models and maximum likelihood estimation to identify groups of individuals that follow similar patterns of behaviour over time. This approach is recommended when it is likely that individuals follow a mixture of distinct patterns of behaviour over time, which is unlikely to be accounted for with a single set of parameters. GBT models are simpler compared to other techniques used to identify latent classes of subjects with comparable growth trajectories, such as growth mixture models, yet they have been shown to outperform them in terms of their ability to identify optimal solutions with fewer classes [36].

In a GBTM framework, the parameters determining each group’s trajectory are the latent growth factors, i.e. intercepts and slopes. The intercept refers to the initial score at baseline and the slope corresponds to the rate of change of the trajectory across assessments. Non-linear trajectories can also be captured by introducing additional quadratic or cubic growth parameters in the model. To identify the optimal number of trajectory groups, models with 2 to 5 groups were tested and compared using goodness of fit criteria including the Bayesian information criterion (BIC) and the log Bayes Factor, whereby a higher BIC (i.e. least negative) and log Bayes factor > 10 represents a better fitting model than the last; the requirement of at least 5% of participants in each trajectory group; close agreement between the estimated probability of group membership and actual the proportion of the sample assigned to that group; posterior probabilities of >0.70; and odds of correct classification based on posterior probabilities exceeding 5 [24]. For each subject, the model provides the probability of belonging to each of the identified trajectory groups and assigns the subject to the trajectory group based on the highest probability. GBTM also allows for the inclusion of time-varying and time-stable predictors simultaneous to class selection in order to increase classification accuracy and to adjust the shape of each group’s trajectory. A multinomial logit function is used to estimate the odds of trajectory group membership according to time-stable predictors, [35] while incorporating time-varying predictors in the model allows for a trajectory to depend on additional variables beyond age or time. The effects of time-varying predictors on the trajectory shapes are estimated for each trajectory group, and thus the effects could differ in each trajectory group. Estimates for time-varying covariates represent the shift in PA trajectory per unit change in the exposure variable. After the optimal number of trajectory groups had been determined, the level of the polynomial function for each group was reduced, starting with quadratic, until each parameter estimate was statistically significant (*p* < 0.05). We considered the following outcomes from the GBT model: 1) the optimal number of trajectory groups, 2) growth parameters describing the shape of each trajectory group 3) the proportion of men assigned to each group, 4) significant predictors of group membership and 5) the effects of time-varying variables on PA slopes by trajectory groups. Trajectories were estimated for men with at least three valid measures of physical activity across the four possible time points accompanied by complete covariate data (*n* = 4952). To understand whether attrition may have affected our results, we also performed a sensitivity analyses identifying trajectories in all men with at least one physical activity measure (*n* = 7646). Finally, as the older men in this cohort transitioned from midlife to old age before the younger men, we also hypothesised that the trajectories of physical activity may be age specific. Thus, in a sensitivity analysis we present trajectories separately for younger and older men.

## Results

**Fig. 1:**
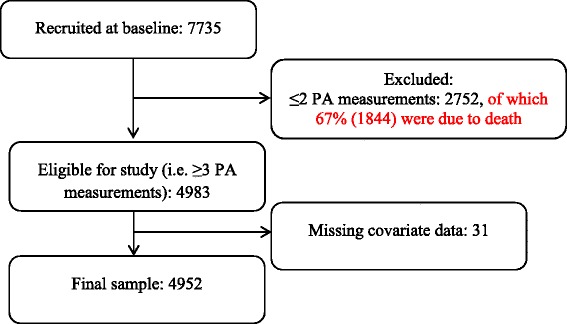
Flow diagram of study participants

Out of the 7735 men originally invited, 2752 (36%) men with <3 PA measurements were excluded from the main analysis. Of the 2752 excluded, 1844 (67%) were lost to follow up due to death. A further 31 (0.4%) were excluded due to missing covariate data. Fig. 1 illustrates a flow diagram of the study participants. Compared to men in the analytic sample, men excluded due to having only 1 or 2 physical activity measures (*n* = 2694) were less active at baseline (53.0% vs. 62.3% classified with at least light activity, respectively), were older (52.2 years vs. 49.1 years, *p* < 0.001), were more likely to come from manual occupations (71.6% vs. 54.3%) and had more health conditions (e.g. 10.7% and 3.2% of men had ≥1 CVD-related at baseline, respectively).

**Fig. 2:**
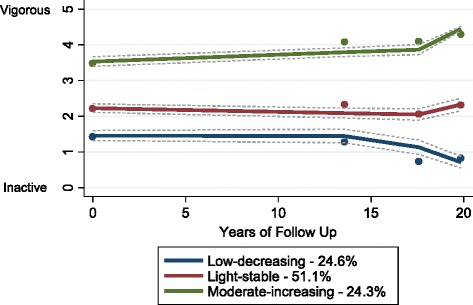
Physical activity trajectories and 95% CIs from midlife to old age (*n* = 4952)

We identified 3 distinct trajectory groups of PA over 20 years of follow up: low decreasing (24.6%, *n* = 1218), light stable (51.1%, *n* = 2530) and moderate increasing (24.3%, *n* = 1204) (see Fig. 2). Although the BIC was higher for the 4 group model, we rejected this model because the difference in BIC was fairly small compared to the 3 group model (see Additional file 1: Table S1). Furthermore, additional groups largely replicated the patterns of the 3 group model but with smaller group sizes. We therefore selected the 3-group model as the most parsimonious description of the longitudinal patterns in the data. In the 3 group model, posterior probabilities ranged from 0.82 to 0.90 and the estimated group sizes were comparable with the actual group sizes, suggesting a good fit. Odds ratios for correct classification ranged from 10.0 to 49.3, suggesting accurate group assignment. Characteristics of the final study sample (*n* = 4952) are presented in Table 1, according to trajectory groups. As expected the proportion of men who were not in employment and with ≥1 CVD-related conditions increased over time across all trajectory groups.

**Table 1:** Participant characteristics at baseline and subsequent follow ups across trajectory groups, mean (SD) or percentage (*n* = 4952, repeated measurements = 17,710)

	Low decreasing (*n* = 1218)				Light stable (*n* = 2530)				Moderate increasing (*n* = 1204)			
	1978/80	1992	1996	2000	1978/80	1992	1996	2000	1978/80	1992	1996	2000
Time-stable												
Age (mean ± SD)	50.4 (5.6)				49.0 (5.6)				48.2 (5.3)			
Manual occupation (%, *n*)	62.7 (764)				57.6 (1457)				38.8 (467)			
Children ≥1 at baseline (%, *n*)	62.9 (766)				71.7 (1815)				73.2 (881)			
Resident in South of England at baseline (%, *n*)	24.0 (292)				36.1 (913)				34.8 (419)			
Overweight/obese at baseline (%, *n*)	60.1 (732)				52.9 (1339)				48.1 (579)			
Married/previously married at baseline (%, *n*)	94.8 (1154)				96.4 (2438)				97.6 (1175)			
Chest pain at baseline (%, *n*)	8.7 (106)				4.6 (115)				3.2 (39)			
Breathlessness at baseline (%, *n*)	9.9 (120)				2.2 (55)				0.7 (8)			
Arthritis at baseline (%, *n*)	13.9 (169)				7.4 (187)				6.6 (79)			
High blood pressure at baseline (%, *n*)	14.7 (179)				9.1 (229)				8.4 (101)			
Bronchitis at baseline (%, n)	22.2 (270)				13.6 (345)				10.6 (128)			
Breakfast cereal consumption ≥2/week at baseline (%, *n*)	34.7 (423)				53.5 (1354)				58.5 (704)			
Alcohol consumption (≥light^a^) (%, *n*)	68.1 (829)				68.5 (1734)				77.3 (931)			
Current smokers (%, n)	54.1 (659)				30.0 (760)				22.9 (276)			
Time-varying												
Completed PA Questionnaire^b^ (*n*)	1204	1150	1126	849	2512	2434	2391	2034	1202	1168	1146	1042
≥ Light Activity^c^ (%, *n*)	34.6 (417)	26.6 (306)	7.1 (80)	8.4 (71)	66.0 (1659)	74.2 (1806)	65.1 (1557)	72.5 (1475)	90.1 (1083)	98.1 (1146)	98.3 (1127)	98.9 (1030)
High sport participation^d^ (%, *n*)	17.8 (217)	7.5 (87)	4.4 (51)	5.5 (47)	40.1 (1014)	33.4 (814)	32.3 (765)	41.3 (844)	80.1 (964)	92.2 (1090)	93.7 (1089)	94.9 (989)
High recreational activity^e^ (%, *n*)	36.0 (437)	26.7 (315)	7.6 (87)	6.5 (55)	58.0 (1465)	61.4 (1517)	39.5 (949)	39.0 (799)	66.1 (796)	74.5 (880)	61.5 (707)	63.4 (663)
High walking^f^ (%, *n*)	19.4 (234)	32.6 (376)	22.1 (259)	31.9 (276)	29.9 (752)	56.6 (1377)	56.0 (1373)	67.5 (1392)	29.3 (353)	59.7 (688)	58.6 (686)	73.0 (766)
Not in employment† (%, *n*)	6.5 (79)	71.6 (836)	83.9 (985)	88.2 (751)	2.9 (73)	56.5 (1390)	71.8 (1748)	81.2 (1656)	1.6 (19)	52.0 (611)	69.0 (806)	79.1 (828)
CVD conditions ≥1† (%, *n*)	5.3 (64)	23.4 (256)	28.5 (334)	32.1 (276)	2.6 (66)	15.5 (366)	19.4 (473)	22.4 (457)	2.5 (30)	12.1 (139)	15.8 (184)	18.6 (194)

^a^light classified as ≥1 unit per week

^b^men with a complete physical activity (PA) score at each follow up

^c^men classified as having at least light physical activity (scoring ≥2)

^d^high sport was classified as reporting at least occasional participation (less than once a month)

^e^high recreational activity was classified as >4 h/weekend on recreational activities

^f^high walking was classified as >20 mins/day

†total number with complete data on employment, CVD conditions and on physical activity types differs to that of the total number with complete PA questionnaire data at each follow up

In the low decreasing group the quadratic function was statistically significant (*p* = <0.001), suggesting a non-linear shape of the PA trajectory (see Table 2). Members of this group were characterized by light/occasional PA at baseline which was stable between baseline and 12-year follow up. Thereafter, this group showed a decreasing pattern between 12- and 16-year follow ups and a subsequent steeper decline towards being inactive at 20-year follow up. In the light stable group, although the slope was significant (*p* = <0.001) suggesting a slight decline, PA levels remained consistently light over time. Finally, in the moderate increasing group the slope, but not the quadratic term (see Additional file 2: Table S2), was positive and statistically significant (*p* < 0.001) suggesting a linear increase in PA over time (see table 2). We also examined the participation rates in specific types of PA across surveys by trajectory group. Notable decreases in sport and recreational activity were observed in the low decreasing group, but walking had increased by 20-year follow up. In the light stable group, sport participation remained stable, but increases in walking and decreases in recreational activity were observed. In the moderate increasing group sport participation was consistently high (>80%) and walking increased over time and recreational activity remained stable.

**Table 2:** Trajectories of physical activity and the effects of time-varying predictors on trajectory shapes, by trajectory group (*n* = 4952)^a^

	Parameter^b^	Estimate	SE	*p* value
Low Decreasing				
	Intercept	1.362	0.052	<0.001
	Linear	0.073	0.015	<0.001
	Quadratic	−0.006	0.001	<0.001
	Time-varying covariates			
	Not employed	−0.306	0.077	<0.001
	N. of CVD diagnoses	−0.408	0.050	<0.001
Light Stable				
	Intercept	2.212	0.042	<0.001
	Linear	−0.011	0.003	<0.001
	Time-varying covariates			
	Not employed	0.324	0.050	<0.001
	N. of CVD diagnoses	−0.118	0.042	0.005
Moderate Increasing				
	Intercept	3.631	0.050	<0.001
	Linear	0.024	0.004	<0.001
	Time-varying covariates			
	Not employed	0.847	0.065	<0.001
	N. of CVD diagnoses	−0.060	0.060	0.313

^a^Estimates for time-varying covariates represent the shift in physical activity trajectory per unit change in exposure variable

^b^Models adjusted for employment status and number of CVD diagnoses as time-varying covariates, and occupational class, marital status, number of children, region, BMI, arthritis, bronchitis, blood pressure, breathlessness, chest pain, smoking status, alcohol consumption and breakfast consumption at baseline

### Time-stable predictors of trajectory group membership

The effects of time-stable covariates on trajectory class membership are shown in Table 3. A number of sociodemographic characteristics were associated with PA trajectories. Being older, residing in the midlands or north of England, being overweight or obese, being a current smoker and suffering from a range of health conditions was associated with reduced odds of belonging to an active trajectory group (light stable/moderate increasing) compared to the low decreasing group. Working in a manual profession was also associated with reduced odds of belonging to the moderate increasing group when compared to the low decreasing group. Furthermore, being married or previously married, having children, drinking alcohol and eating breakfast cereal was associated with increased odds of belonging to an active trajectory group compared to the low decreasing group.

**Table 3:** Time-stable predictors of trajectory class membership (*n* = 4952)

	Light Stable vs. Low Decreasing^a^		Moderate Increasing vs. Low Decreasing^a^	
	OR (95% CI)^b^	*p* value	OR (95% CI)^b^	*p* value
Socio-demographic factors				
Baseline age (per year increase)	0.97 (0.95, 0.98)	<0.001	0.94 (0.92, 0.96)	<0.001
Occupational class				
Manual (ref. non-manual)	1.10 (0.88, 1.37)	0.401	0.60 (0.48, 0.74)	<0.001
Marital status				
Married (ref. single)	1.41 (0.84, 2.39)	0.197	2.14 (1.22, 3.75)	0.008
Widowed/Divorced (ref. single)	1.98 (0.96, 4.08)	0.064	2.58 (1.20, 5.55)	0.015
Number of children				
≥1 child (ref. no children)	1.42 (1.13, 1.78)	0.003	1.45 (1.15, 1.81)	0.001
Region				
Midlands (ref. south)	0.67 (0.49, 0.92)	0.014	0.68 (0.49, 0.94)	0.018
North (ref. south)	0.71 (0.55, 0.91)	0.008	0.70 (0.55, 0.90)	0.005
Scotland (ref. south)	0.71 (0.49, 1.04)	0.076	1.25 (0.88, 1.78)	0.210
Health and Lifestyle Factors				
Overweight/Obese (ref. healthy BMI)	0.79 (0.64, 0.98)	0.031	0.66 (0.54, 0.81)	<0.001
Arthritis (ref. no arthritis)	0.60 (0.44, 0.83)	0.002	0.54 (0.38, 0.76)	<0.001
Bronchitis (ref. no bronchitis)	0.74 (0.57, 0.97)	0.026	0.62 (0.47, 0.82)	<0.001
High blood pressure (ref. normal blood pressure)	0.71 (0.52, 0.97)	0.034	0.67 (0.48, 0.92)	0.014
Suffers breathlessness (ref. no breathlessness)	0.30 (0.19, 0.48)	<0.001	0.14 (0.06, 0.29)	<0.001
Suffers chest pain (ref. no chest pain)	0.74 (0.48, 1.13)	0.159	0.64 (0.40, 1.02)	0.061
Current/ex-smoker (ref. non-smoker)	0.42 (0.34, 0.52)	<0.001	0.31 (0.24, 0.39)	<0.001
Alcohol consumption				
Occasional (ref. none)	1.29 (0.83, 2.02)	0.260	1.47 (0.89, 2.43)	0.133
Light (ref. none)	1.50 (0.96, 2.35)	0.075	2.73 (1.67, 4.45)	<0.001
Moderate (ref. none)	1.27 (0.81, 1.99)	0.294	2.15 (1.31, 3.53)	0.003
Heavy (ref. none)	1.55 (0.92, 2.61)	0.099	2.30 (1.30, 4.07)	0.004
Dietary habits				
Occasional breakfast cereal (ref. none)	2.13 (1.49, 3.05)	<0.001	2.13 (1.50, 3.03)	<0.001
Regular breakfast cereal (ref. none)	1.57 (1.25, 1.98)	<0.001	1.66 (1.32, 2.08)	<0.001

^a^Low decreasing (reference group). ^b^Models adjusted for employment status and number of CVD diagnoses as time-varying covariates, and occupational class, marital status, number of children, region, BMI, arthritis, bronchitis, blood pressure, breathlessness, chest pain, smoking status, alcohol consumption and breakfast consumption at baseline

### Time-varying predictors of trajectory slopes

The associations between time-varying covariates and PA in each trajectory group are presented in Table 2. Leaving employment was associated with a decrease in PA in the low decreasing group (β −0.306, *p* < 0.001) but an increase in the light stable (β 0.324, *p* < 0.001) and moderate increasing groups (β 0.847, *p* < 0.001). The primary reason for leaving employment was retirement (i.e. 86%, 94% and 97% of men not in employment had retired at 12, 16 and 20-year follow ups, respectively). Further analysis revealed a higher proportion of men in the low decreasing group were retiring due to health issues (30%) compared to the light stable (15%) and moderate increasing groups (11%). Development of cardiovascular-related conditions was associated with a decrease in PA in the low decreasing (β −0.408, *p* < 0.001) and light stable groups (β −0.118, *p* < 0.001) but no association was observed in the moderate increasing group (β −0.060, *p* = 0.313). Wald tests revealed that the effects of employment status and CVD diagnoses were significantly different between trajectory groups (*p* < 0.001). In a supplementary analysis, we included alcohol consumption and smoking as time-varying covariates. This analysis showed that an increase in alcohol consumption was associated with an increase in PA in the active trajectory groups, while quitting smoking was associated with an increase in PA across all trajectory groups (Additional file 3: Table S3).

### Sensitivity analyses

Incorporating all available data into the trajectory group analysis (*n* = 7646), which included men with PA data at one or two assessments (*n* = 2694), the three group model remained optimal and the shapes of the trajectories remained largely unchanged. Associations with exposure variables also remained unchanged. However, group sizes were altered, with a higher proportion of men belonging to the low decreasing trajectory group (34.3%) and a lower proportion in the moderate increasing trajectory group (18.7%). This suggests that PA levels were lower in men who were lost to follow up and that attrition may have led to an overestimation of PA levels. However, this is unlikely to have affected the trajectory shapes and optimal number of groups. When we stratified our sample into younger (mean age at baseline, 45.1 ± 3.0) and older men (mean age at baseline, 54.8 ± 2.8), the results remained largely unchanged; however, this revealed important periods of change that were previously masked. The early sixties emerged as a period in the lifecourse when PA may be more likely to increase (see Additional file 4: Figure S1); however, PA may start to decline as men approach their late sixties/early seventies.

## Discussion

We identified 3 distinct trajectories of PA in men transitioning from midlife to old age: low decreasing, light stable and moderate increasing. Men following light stable and moderate increasing trajectories had fewer health conditions, generally engaged in other healthy behaviours and had more advantaged social background characteristics in midlife than men following low decreasing trajectories. Furthermore, the effects of developing CVD-related conditions and changing employment status varied according to trajectory groups.

### Relation to previous studies

Other recent studies, using similar statistical approaches [15, 37, 38], have also identified a large proportion of older adults who are persistently inactive [15]. However, such studies have typically included groups that experience large declines and/or increases in PA [15, 37, 38]. The results of the present study suggest that although PA levels change during the transition to old age, PA levels in old age are largely determined by PA levels in midlife or earlier. These discrepancies may at least partly reflect differences in the reporting instruments and cut offs used to measure PA. For example, one of the aforementioned studies examined 11-year PA trajectories in a cohort of Taiwanese older adults and used a binary variable, derived from self-reported sport and exercise participation [15]. Another study from the Women’s Health and Aging Study II used a categorical variable, classifying women as inactive, moderately active, and very active based on self-reported data [38]. Objective measures of PA that were previously unavailable when many of these cohort studies began, may provide a more accurate estimate of total time spent in PA. Although in the present study PA was measured subjectively, a key strength is that the PA questionnaire covers several types of PA. As such, we were able to observe how different types of PA contribute to these trajectories. In the low-decreasing group, declines were largely due to decreases in sport and recreational activity; however, interestingly, walking actually increased. Interventions targeting the most inactive older age members of the population may benefit from promoting walking as this group appear to be more likely to take up this type of PA.

### Predictors of trajectory patterns

The present study identified a number of sociodemographic factors associated with membership of more ‘active’ trajectory groups (light stable or moderate increasing). For example, being older, coming from manual occupations, not being married and being resident in the Midlands or North of England was associated with reduced odds of following an active trajectory. Previous studies have found that older adults are more inactive [9] and more likely to follow inactive trajectories over time [14] compared to younger counterparts. Further, socio-economic disadvantage, of which occupation class is closely related to, has also been associated with lower PA. [14, 15, 39] Our results suggest socioeconomic disadvantage may predict long-term inactivity into old age. Current evidence on the association between marital status and PA in older adults has been somewhat mixed. [15, 37, 40] One study in adults aged 70–79 showed that married men had higher levels of exercise than single men of the same age [40]. There is also evidence for close concordance between the PA trajectories of married couples. [37] Plausibly, being married may prevent isolation in old age and provide a companion for physical activities. On the other hand, having a spouse may involve care-giving responsibilities, which may prove to be a barrier for maintaining or increasing PA levels. [15]. Regional differences in PA levels in England have previously been reported in cross-sectional studies [41], with higher prevalence of inactivity reported in adults from Northern England compared to adults from Southern regions of England. In our study, we show that these regional trends persist over long periods in adults transitioning to old age. Regional differences in PA levels may be explained by variation in the social and built environment and access to leisure facilities; however, further research is required to fully understand this regional variation.

Men with children were also more likely to be members of active trajectory groups compared to the low decreasing group. There is a large body of evidence demonstrating the influence of parental behaviours on children’s PA levels [42] but very little research has been conducted on the impact of having children on the parent’s long-term PA levels. From the limited studies available it has been shown that parenthood is associated with an acute decrease in PA levels in mothers [43, 44]; however, the present study demonstrates a positive long-term impact of fatherhood on PA into old age. A larger family network including children and possibly grandchildren may facilitate a more active lifestyle through more social activities and increased childcare responsibilities. Higher PA trajectories may also be one pathway explaining the observed association between parenthood and increased longevity [45].

As expected, men suffering from a variety of health problems at baseline were less likely to be members of an active trajectory group compared to the low decreasing group. These health conditions included being overweight or obese; diagnosed arthritis, bronchitis and high blood pressure; chest pain; and breathlessness. Previous studies have highlighted worse overall health as a predictor of declines in PA in older adults [46], but few have quantified the impact of specific conditions. In the present study, breathlessness in midlife was the strongest health-related predictor of an inactive trajectory into old age. Overall, early onset of health conditions, particularly breathlessness, may represent a major barrier for maintenance of a physically active lifestyle into old age.

Men who did not smoke and who consumed breakfast cereal were also more likely to be members of an active trajectory group compared to the low decreasing group. This may reflect the tendency for healthy lifestyle behaviours to cluster. For example, skipping breakfast is associated with the clustering of other health-compromising behaviours such as sedentary behaviour, smoking and alcohol use [47]. Alternatively, it is also possible that breakfast consumption provides energy for morning PA and facilitates PA readiness [48, 49]. Breakfast habits in the elderly may be particularly important given that older adults are generally most active in the mornings [50]. Interestingly, not all healthy behaviours were associated with following an active trajectory. In contrast to dietary and smoking habits, men consuming greater amounts of alcohol were more likely to be members of the moderate increasing trajectory group. This is consistent with a recent systematic review that found a positive association between PA and alcohol consumption [51]. One hypothesis is that PA creates more social activities and therefore more opportunities for alcohol consumption. Alternatively, adults who drink alcohol may seek to compensate for this unhealthy behaviour by performing more PA [52]. Further research is required to understand the mechanisms that link PA levels with alcohol consumption, but independent strategies to tackle alcohol use problems in active older adults may be required.

Overall, the associations between time-stable covariates and trajectory group membership were in the expected direction. However, these results extend on what is already known by highlighting factors that predict long term PA. This information might be useful for interventions targeting individuals who are most at risk of long-term inactivity into old age.

### Influence of time-varying predictors on trajectory slopes

Leaving employment was associated with increased PA in the active trajectory groups but decreased PA in the low-decreasing group. The primary reason for leaving employment was retirement, suggesting that retirement may be associated with an increase in PA in men who are already at least occasionally active. Previous research has also indicated that retirement can lead to increases in leisure time PA [20–23], possibly due to increased free time. However, our results suggest that this association may be limited to individuals who are already partaking in some PA prior to retirement. Retiring whilst free from disease may be an important mediator of this association. Members of the low-decreasing group typically came from manual occupations, suggesting that the majority of their PA was work related. Hence, retirement results in PA declines in this group. Different strategies may be required to increase PA in retiring men who are already inactive or coming from manual professions.

An increase in the number of diagnosed CVD conditions was associated with a decline in PA levels in the light stable and low decreasing groups. PA has been well established as a protective behaviour against CVD [1] but few studies have quantified how CVD is associated with changes in PA over prolonged periods. Onset of CVD conditions may reduce the functional capacity to carry out PA in older adults, particularly in those who have lower PA levels to begin with. However, there is evidence that functional and aerobic capacity can recover to nearly normal levels after CVD events, such as heart attack or stroke, even without intervention [53, 54]. Individuals who are active prior to a cardiac event may be preconditioned with greater tolerance to such events and subsequently have a better recovery. For example, higher pre-stroke PA levels have been associated with improved physical function and greater expression of vascular endothelial growth factors after stroke [55, 56]. The present study extends on this research by showing this tolerance exists in active community-dwelling older adults and that it persists over prolonged periods. Cardiac rehabilitation services may need to target individuals who were inactive prior to their cardiac event.

### Strengths and limitations

The main strength of this study is the data-driven approach for identifying PA trajectories. This technique also allowed us to examine the effects of both time-stable and time-varying predictors on PA trajectories. Unfortunately, we were unable to capture the effects of all variables that could potentially vary over time due to data availability. Some variables may have had dynamic effects on PA that we were unable to capture. More prospective research capturing time-varying data will help unearth how other events, such as onset of other health conditions, influence PA trajectories. This study is also limited by the use of self-reported PA, which may have been prone to recall bias. However, the questionnaire was validated at baseline against heart rate and forced expiratory volume in 1 s and more recently against objectively measured PA [33, 34]. Self-reported data also provide an insight into the types of PA that contribute to these trajectories, which may help inform intervention strategy. Although the 20-year trajectories cover a significant proportion of the lifespan, important fluctuations in PA levels may have been missed in the 12-year interval between baseline and the next follow up assessment. Other important periods prior to midlife may be critical for lifelong PA trajectories but were not captured in this study. PA habits formed earlier in life may be associated with an increased risk of chronic conditions in midlife. Thus we cannot ignore the possibility of reverse causation. There is also the possibility that men dropping out of the study may have affected our trajectories. Sensitivity analyses revealed that the proportion of men who follow a low decreasing trajectory may have been underestimated owing to men who were lost to follow up being less active; however, this is unlikely to have affected the number of trajectories and trajectory shapes as they were comparable when we included men with ≥1 time point rather than requiring ≥3 time points. An important advantage of using the maximum likelihood method, which is incorporated into the GBTM framework, is that it is able to utilise all available data to estimate parameters and standard errors, under the assumption that missingness is at random. Lastly, another important limitation is that our sample were predominantly white males, which means our findings may not be generalizable to women and non-white ethnic groups.

## Conclusion

Three distinct trajectories of PA from midlife to old age were identified, with almost a quarter of the sample being categorised as essentially persistently inactive. PA levels in old age were largely dictated by PA in midlife. Interventions targeting adults transitioning into old age may need to consider a number of sociodemographic characteristics, family features and health and lifestyle factors. Strategies focussing on promoting PA in adults who are retiring or recovering from a cardiovascular event may need to be tailored according to prior PA.

## Additional files

Additional file 1: Table S1. Model search process for physical activity trajectories (*n* = 4952) (DOCX 15 kb)

Additional file 2: Table S2. Determining the highest model function of the 3 physical activity trajectory groups (n = 4952) (DOCX 14 kb)

Additional file 3: Table S3. Trajectories of physical activity and the effects of time-varying predictors on trajectory shapes, by trajectory group (*n* = 4962) (DOCX 15 kb)

Additional file 4: Figure S1. Physical activity trajectories and 95% CIs from midlife to old age stratified according to baseline age (n=4952). (DOCX 31 kb)

## Abbreviations

BIC: Bayesian information criterion
BMI: Body mass index
CVD: Cardiovascular disease
GBTM: Group-based trajectory modelling
PA: Physical activity
