# Supplementary material for: Trajectories of self-reported physical activity and predictors during the transition to old age: a 20-year cohort study of British men
Source: Int J Behav Nutr Phys Act. 2018 Feb 7;15:14. doi: 10.1186/s12966-017-0642-4 (PMC5803992; doi:10.1186/s12966-017-0642-4)
Supplement: Supplementary file 1 — Model search process for physical activity trajectories (n = 4952) (DOCX 15 kb) [file 12966_2017_642_MOESM1_ESM.docx]

**Additional file 1: Table S1.** Model search process for physical activity trajectories (n=4952)

| **Number of groups** | | **BIC** | **Log Bayes Factor (2*ΔBIC)** | | **Estimated group %** | | | **Actual group %** | | **Posterior probability** | |  |  |  |  |  |
| --- | --- | --- | --- | --- | --- | --- | --- | --- | --- | --- | --- | --- | --- | --- | --- | --- |
|  | |  |  | | 66.6 | | | 67.2 | | 0.95 | |  |  |  |  |  |
| 2 | | -30739.6 |  | | 33.4 | | | 32.8 | | 0.92 | |  |  |  |  |  |
|  | |  |  | | 26.0 | | | 24.3 | | 0.82 | |  |  |  |  |  |
|  | |  |  | | 49.4 | | | 51.4 | | 0.83 | |  |  |  |  |  |
| 3 | | -30442.9 | 593.4 | | 24.6 | | | 24.3 | | 0.90 | |  |  |  |  |  |
|  | |  |  | | 16.7 | | | 14.3 | | 0.80 | |  |  |  |  |  |
|  | |  |  | | 50.1 | | | 53.5 | | 0.84 | |  |  |  |  |  |
|  | |  |  | | 26.8 | | | 26.2 | | 0.85 | |  |  |  |  |  |
| 4 | | -30385.6 | 114.6 | | 6.4 | | | 6.0 | | 0.84 | |  |  |  |  |  |
|  | |  |  | | 5.9 | | | 6.3 | | 0.73 | |  |  |  |  |  |
|  | |  |  | | 26.6 | | | 24.1 | | 0.71 | |  |  |  |  |  |
|  | |  |  | | 37.5 | | | 39.8 | | 0.72 | |  |  |  |  |  |
|  | |  |  | | 24.7 | | | 24.5 | | 0.84 | |  |  |  |  |  |
| 5 | | -30458.9 | -146.6 | | 5.4 | | | 5.3 | | 0.84 | |  |  |  |  |  |
|  |  |  |  |  |  |  |  |  |  |  |  |  |  |  |  |  |
|  | |  | |  | |  |  | |  | |  | |  |  |  |  |

BIC, Bayesian information criterion.

Models adjusted for employment status and number of CVD diagnoses as time-varying covariates, and occupational class, marital status, number of children, region, BMI, arthritis, bronchitis, blood pressure, breathlessness, chest pain, smoking status, alcohol consumption and breakfast consumption at baseline.
