# Supplementary material for: Trajectories of self-reported physical activity and predictors during the transition to old age: a 20-year cohort study of British men
Source: Int J Behav Nutr Phys Act. 2018 Feb 7;15:14. doi: 10.1186/s12966-017-0642-4 (PMC5803992; doi:10.1186/s12966-017-0642-4)
Supplement: Supplementary file 2 — Determining the highest model function of the 3 physical activity trajectory groups (n = 4952) (DOCX 14 kb) [file 12966_2017_642_MOESM2_ESM.docx]

**Additional file 2: Table S2.** Determining the highest model function of the 3 physical activity trajectory groups (n=4952) ^a,b^

|  | **1^st^ iteration** | | **2^nd^ iteration** | |  | |  | |  |
| --- | --- | --- | --- | --- | --- | --- | --- | --- | --- |
| **Group** | **Highest function** | ***p*** | **Highest function** | ***p*** | | **Final estimated group %** | | **Final actual group %** | |
| Group 1 | Quadratic | <0.001 | Quadratic | <0.001 | | 26.3 | | 24.6 | |
| Group 2 | Quadratic | 0.323 | Linear | <0.001 | | 49.2 | | 51.1 | |
| Group 3 | Quadratic | 0.246 | Linear | <0.001 | | 24.5 | | 24.3 | |

^a^Starting with quadratic, the level of the polynomial function for each group was reduced at each iteration until each parameter estimate was statistically significant (p<0.05). ^b^Models adjusted for employment status and number of CVD diagnoses as time-varying covariates, and occupational class, marital status, number of children, region, BMI, arthritis, bronchitis, blood pressure, breathlessness, chest pain, smoking status, alcohol consumption and breakfast consumption at baseline.
