# Supplementary material for: Trajectories of self-reported physical activity and predictors during the transition to old age: a 20-year cohort study of British men
Source: Int J Behav Nutr Phys Act. 2018 Feb 7;15:14. doi: 10.1186/s12966-017-0642-4 (PMC5803992; doi:10.1186/s12966-017-0642-4)
Supplement: Supplementary file 3 — Trajectories of physical activity and the effects of time-varying predictors on trajectory shapes, by trajectory group (n = 4962) (DOCX 15 kb) [file 12966_2017_642_MOESM3_ESM.docx]

Additional file 3: Table S3. Trajectories of physical activity and the effects of time-varying predictors on trajectory shapes, by trajectory group (n=4962)^a^

|  | **Parameter^b^** | **Estimate** | **SE** | **p value** |
| --- | --- | --- | --- | --- |
| Low Decreasing |  |  |  |  |
|  | Intercept | 1.216 | 0.096 | <0.001 |
|  | Linear | 0.060 | 0.014 | <0.001 |
|  | Quadratic | -0.005 | 0.001 | <0.001 |
|  | Time-varying covariates |  |  |  |
|  | Not employed | -0.278 | 0.075 | <0.001 |
|  | N. of CVD diagnoses | -0.416 | 0.048 | <0.001 |
|  | Alcohol consumption† | 0.022 | 0.024 | 0.365 |
|  | Quitting smoking | 0.220 | 0.060 | <0.000 |
| Light Stable |  |  |  |  |
|  | Intercept | 1.780 | 0.079 | <0.001 |
|  | Linear | -0.011 | 0.003 | <0.001 |
|  | Time-varying covariates |  |  |  |
|  | Not employed | 0.359 | 0.054 | <0.001 |
|  | N. of CVD diagnoses | -0.121 | 0.045 | 0.007 |
|  | Alcohol consumption† | 0.080 | 0.020 | <0.001 |
|  | Quitting smoking | 0.331 | 0.051 | <0.001 |
| Moderate Increasing |  |  |  |  |
|  | Intercept | 3.233 | 0.125 | <0.001 |
|  | Linear | 0.023 | 0.004 | <0.001 |
|  | Time-varying covariates |  |  |  |
|  | Not employed | 0.831 | 0.068 | <0.001 |
|  | N. of CVD diagnoses | -0.061 | 0.063 | 0.334 |
|  | Alcohol consumption† | 0.058 | 0.032 | 0.069 |
|  | Quitting smoking | 0.343 | 0.074 | <0.001 |

^a^Estimates for time-varying covariates represent the shift in physical activity trajectory per unit change in exposure variable.

^b^Models adjusted for employment status and number of CVD diagnoses as time-varying covariates, and occupational class, marital status, number of children, region, BMI, arthritis, bronchitis, blood pressure, breathlessness, chest pain, smoking status, alcohol consumption and breakfast consumption at baseline

†Alcohol was entered as a 5 point categorical variable (none, occasional [<1 drink/week], light [1-15 drinks/week], moderate [16-42 drinks/week] or heavy [>42 drinks/week]).
