# Supplementary material for: Trajectories of self-reported physical activity and predictors during the transition to old age: a 20-year cohort study of British men
Source: Int J Behav Nutr Phys Act. 2018 Feb 7;15:14. doi: 10.1186/s12966-017-0642-4 (PMC5803992; doi:10.1186/s12966-017-0642-4)
Supplement: Supplementary file 4 — Physical activity trajectories and 95% CIs from midlife to old age stratified according to baseline age (n=4952). (DOCX 31 kb) [file 12966_2017_642_MOESM4_ESM.docx]

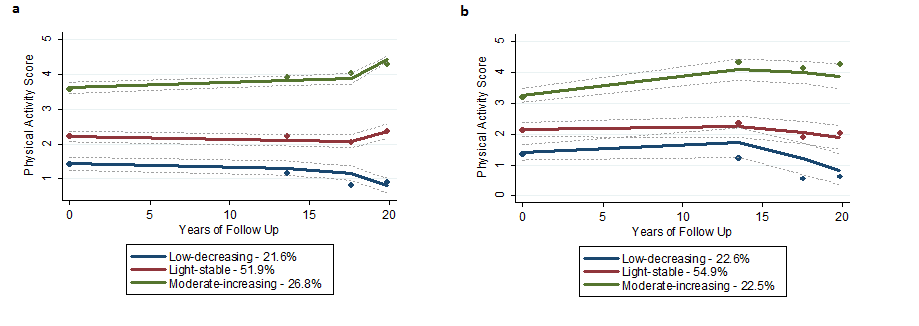


A. Physical activity trajectories and 95% CIs in younger men B. Physical activity trajectories and 95% CIs in older men

(mean age at baseline, 45.1 ± 3.0) (mean age at baseline, 54.8 ± 2.8)
